# Aortic Dissection in c-ANCA-Associated Vasculitis: A Case Report and Literature Review

**DOI:** 10.7759/cureus.84420

**Published:** 2025-05-19

**Authors:** Sidhartha G Senapati, Joel Shah, Lakshmi Kattamuri, Angelica Lehker

**Affiliations:** 1 Internal Medicine, Texas Tech University Health Sciences Center El Paso, El Paso, USA

**Keywords:** anti-proteinase 3 antibodies, aortic dissection, c-anca vasculitis, granulomatosis with polyangiitis (gpa), wegener’s granulomatosis

## Abstract

Cytoplasmic antineutrophil cytoplasmic antibodies (c-ANCA)-associated vasculitis usually affects medium-sized and small vessels, with aortic involvement extremely rare. While vasculitis commonly leads to systemic inflammation, large vessel complications like aortitis or dissection are less well-documented. A male in his 50s with hypertension presented with sudden-onset, severe chest pain radiating to his back and shortness of breath. Pain slightly improved with opioids. Initial CT showed moderate thickening of the thoracic aorta, suggestive of vasculitis. High-dose steroids were started. Autoimmune tests confirmed elevated c-ANCA and anti-proteinase 3 antibodies. A follow-up CT angiography later revealed a Stanford type A aortic dissection extending from the thoracic to the abdominal aorta, involving the superior mesenteric artery and inferior mesenteric artery. The patient was urgently transferred for surgical intervention. Initial treatment with steroids was based on suspected vasculitis. The discovery of a Stanford type A aortic dissection required urgent surgical repair. Timely imaging was critical in identifying this severe complication, leading to rapid transfer for surgery. This case highlights the rare but serious possibility of aortic involvement in c-ANCA-associated vasculitis. It underscores the importance of serial imaging to detect and manage severe complications early, emphasizing adaptable treatment strategies for better outcomes.

## Introduction

Antineutrophil cytoplasmic antibodies (ANCA)-associated vasculitis (AAV) encompasses autoimmune disorders primarily affecting small to medium-sized blood vessels, including granulomatosis with polyangiitis (GPA) and microscopic polyangiitis (MPA) [1]. GPA, characterized by necrotizing granulomatous inflammation, typically involves the respiratory tract and kidneys, with 80-90% of patients exhibiting proteinase 3 ANCA (PR3-ANCA, also called cytoplasmic ANCA or c-ANCA), which drives granulomatous tissue damage [2]. MPA, marked by non-granulomatous vasculitis, targets the kidneys and lungs, often associated with myeloperoxidase ANCA (MPO-ANCA), linked to neutrophilic vessel wall necrosis [2]. GPA has an estimated incidence of 10-20 per million annually and follows a relapsing-remitting course [3]. Untreated, GPA carries a 70% one-year mortality rate; modern treatments (e.g., corticosteroids and rituximab) achieve remission in over 80% of cases, though relapses occur in ~50% [4]. PR3-ANCA-positive patients may have higher relapse rates than MPO-ANCA-positive cases, with similar mortality [3].

Although AAV predominantly affects small vessels, large-vessel involvement, such as aortitis or dissection, occurs in fewer than 10% of cases, with aortic complications estimated at less than one per 1000 AAV patients annually [5,6]. Aortic involvement has been reported in GPA and MPA but not in eosinophilic granulomatosis with polyangiitis (EGPA), likely due to EGPA’s eosinophilic, allergic inflammatory profile [5]. In GPA and MPA, PR3-ANCA or MPO-ANCA-mediated neutrophil activation causes aortitis by releasing cytokines (e.g., tumor necrosis factor (TNF)-alpha and IL-1) and matrix metalloproteinases (MMPs) that degrade elastin and collagen in the aortic media, weakening the vessel wall [5,7,8]. Granulomatous inflammation and endothelial dysfunction further disrupt aortic integrity, predisposing to dissection under mechanical stress (e.g., hypertension) [7,8]. Chronic aortitis is rare in AAV compared to large-vessel vasculitides like Takayasu arteritis, contributing to underdiagnosis due to atypical presentations [8,9].

Aortic dissection, a life-threatening condition involving a tear in the aortic inner layer, is severe in AAV, with up to 50% mortality without urgent surgery [5]. Approximately 16 cases of AAV-related aortic dissection have been reported, primarily in men with acute chest pain, often in newly diagnosed GPA, based on case reports [7,9,10]. Increasing recognition may reflect improved imaging, such as high-resolution computed tomography angiography (CTA) [7,10]. Risk factors include hypertension and active vasculitis [7,9,10]. For example, Chirinos et al. described a 53-year-old woman misdiagnosed with pneumonia, later found to have MPA-related fatal aortic dissection [5]. In contrast, this case involves rapid progression from aortitis to Stanford type A dissection within 48 hours in a newly diagnosed GPA, successfully managed with surgery despite chronic low-dose prednisone use for Crohn’s disease, highlighting the need for early detection using CTA and PR3-ANCA testing to guide rituximab and surgical intervention [2,5,11]. This report explores diagnostic and management challenges in GPA-related aortic dissection.

## Case presentation

Clinical presentation and symptoms

A man in his 50s presented to the emergency department with acute, severe chest pain, sudden in onset and maximal at the start, described as mid-sternal with a burning sensation, radiating to the back, spine, and both legs, accompanied by dyspnea and cold sweats lasting two to three minutes. Over the past month, he reported intermittent cold and blue discoloration of hands and feet during physical activity, without digital ischemia, livedo reticularis, or vasculopathic changes. He denied classic Raynaud’s phenomenon (e.g., triphasic color changes triggered by cold), though vasculitis-related digital ischemia may present atypically. Key red flags included a pulse deficit (absent bilateral radial and left posterior tibial pulses) and severe hypertension (blood pressure 210/114 mmHg bilaterally, no inter-arm discrepancy). No murmurs suggestive of aortic regurgitation were noted. His medical history included hypertension (12 years, well-controlled with hydralazine 50 mg twice daily) and Crohn’s disease (five years, managed with prednisone 20 mg daily and ustekinumab injections). He had no family history of aortic aneurysms or connective tissue disorders. Risk factors for aortic dissection included chronic inflammation from suspected GPA and long-term low-dose prednisone, with no prior vascular involvement documented. He was a lifelong non-smoker and denied visual disturbances, rashes, hair loss, fevers, weight loss, melena, or hematochezia. Opioid analgesics provided partial pain relief.

Physical examination

Blood pressure was 210/114 mmHg bilaterally. Cardiovascular exam showed regular rate and rhythm, with no murmurs, rubs, or gallops. Respiratory exam indicated clear lung fields. Abdominal exam was unremarkable, with no tenderness, masses, or bruits. Bilateral radial and left posterior tibial pulses were absent, but no acute ischemic changes (e.g., pallor, coolness, and motor deficits) suggested chronic vascular involvement with collateral perfusion. A bedside funduscopic exam was normal, and no rashes, joint swelling, or neurological deficits were observed.

Diagnostic investigations

Workup included an electrocardiogram (ECG), showing no ischemic changes, and normal cardiac troponins, ruling out acute coronary syndrome. Laboratory tests revealed hemoglobin at 9.4 g/dL (normal: 13.5-17.5 g/dL), suggesting chronic inflammation; erythrocyte sedimentation rate (ESR) of 77 mm/hr (normal: 0-20 mm/hr), indicating significant inflammation; C-reactive protein (CRP) at 5.53 mg/dL (normal: <0.5 mg/dL), supporting active vasculitis; cytoplasmic ANCA (c-ANCA) at 1:20 (normal: <1:16), supportive but not specific for GPA; proteinase 3 ANCA (PR3-ANCA) of 4.3 AI (normal: <1.0), highly specific for GPA; negative antinuclear antibody (ANA), rheumatoid factor, and perinuclear ANCA (p-ANCA), ruling out drug-induced lupus or other vasculitides; red cell distribution width (RDW) of 9.6% (normal: 11.5-14.5%), likely a lab artifact, as no clinical correlation exists; mean platelet volume (MPV) of 16.3 fL (normal: 7.5-11.5 fL), possibly reflecting reactive thrombocytosis; serum creatinine at 0.8 mg/dL (normal: 0.6-1.2 mg/dL), indicating no renal involvement (Table 1).

**Table 1:** Laboratory investigations with normal reference ranges. MCV: mean corpuscular volume; MCH: mean corpuscular hemoglobin; MCHC: mean corpuscular hemoglobin concentration; RDW: red cell distribution width; MPV: mean platelet volume; CRP: C-reactive protein; ESR: erythrocyte sedimentation rate; CK-MB: creatine kinase-myocardial band; RPR: rapid plasma reagin; ANA: antinuclear antibody; c-ANCA: cytoplasmic antineutrophil cytoplasmic antibodies; p-ANCA: perinuclear antineutrophil cytoplasmic antibodies; ALT: alanine aminotransferase; AST: aspartate aminotransferase.

Category	Parameter	Value	Normal range
Complete blood count	WBC (x10^9/L)	9.14	4.0-11.0
	RBC (x10^12/L)	3.31	4.5-5.9 (males), 4.1-5.1 (females)
	Hemoglobin (g/dL)	9.4	13.8-17.2 (males), 12.1-15.1 (females)
	Hematocrit (%)	31.2	40-52 (males), 35-47 (females)
	MCV (fL)	89.4	80-100
	MCH (pg)	25.7	27-33
	MCHC (g/dL)	28.7	31-36
	Platelets (x10^9/L)	265	15-450
	RDW (%)	9.6	11.5-14.5
	MPV (fL)	16.3	7.5-12.0
Differential	Neutrophils (%)	7.97	40-70
	Eosinophils (%)	0.50	0-6
	Basophils (%)	0.59	0-2
	Monocytes (%)	0.00	2-10
	Lymphocytes (%)	87.1	20-40
	Atypical lymphocytes (%)	6.5	<1
Chemistry routine	Sodium (mmol/L)	137	135-145
	Potassium (mmol/L)	5.0	3.5-5.1
	Chloride (mmol/L)	103	98-106
	Bicarbonate (mmol/L)	26	22-29
	Urea (mg/dL)	8	7-20
	Creatinine (mg/dL)	0.80	0.6-1.2
	Glucose (mg/dL)	105	70-99
	Calcium (mg/dL)	9.2	8.5-10.2
	Phosphate (mg/dL)	2.6	2.5-4.5
	Magnesium (mg/dL)	3.3	1.7-2.2
	Albumin (g/dL)	6.2	3.5-5.0
	CRP (mg/L)	5.53	<5
	ESR (mm/hr)	77	-
Cardiac markers	CK-MB (ng/mL)	0.022	0-3.6
Serology	RPR	Non-reactive	Non-reactive
	ANA	Negative	Negative
	COVID-19 rapid	Negative	Negative
Miscellaneous	DNase-B antibody	Negative	Negative
	c-ANCA	1:20	<1.20
	Proteinase-3 antibody (AI)	4.3	2.0
	p-ANCA	Negative	Negative
Enzymes & cardiac markers	ALT (U/L)	17	7-56
	AST (U/L)	53	10-40

**Figure 1:**
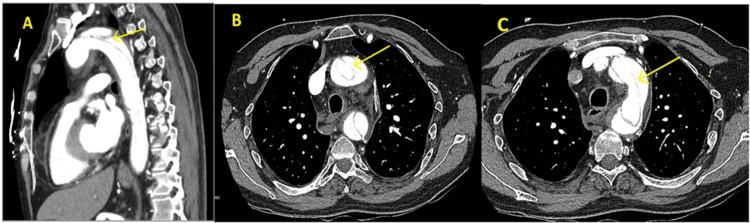
CT angiogram of the chest with contrast depicting Stanford type A dissection of the aorta (as indicated by the arrow).

Testing for GPA was prompted within hours by red flags (chest pain, pulse deficits, hypertension), elevated ESR/CRP, and an initial contrast-enhanced CT scan (day zero) showing diffuse, circumferential aortic wall thickening without perivascular inflammation, vessel wall enhancement, or luminal narrowing, consistent with aortitis. Differential diagnoses, including Takayasu arteritis (ruled out by age, serology, lack of luminal narrowing) and Crohn’s-related vasculitis (ruled out by PR3-ANCA positivity, no gastrointestinal flares), were excluded. High-dose intravenous methylprednisolone (1 g daily) was initiated on day one for suspected GPA. A follow-up CTA on day two (~48 hours after initial CT), prompted by persistent chest pain and worsening dyspnea, confirmed a Stanford type A aortic dissection extending from the ascending aorta to the abdominal aorta, involving the right brachiocephalic, right common carotid, left common carotid, left subclavian, superior mesenteric artery (SMA), and inferior mesenteric artery (IMA) (Figure 1). No malperfusion syndromes (e.g., stroke and limb ischemia) were evident.

Disease progression and management

Rapid progression from aortitis (day one CT) to dissection (day two CTA) prompted urgent vascular surgery consultation. The patient was sent directly for surgery on day two, with no additional workup (e.g., transesophageal echocardiography) due to clear CTA findings and clinical urgency. Emergency surgical repair (ascending aorta and hemiarch replacement with a Dacron graft) was performed, with intraoperative findings confirming aortitis. Postoperatively, the patient stabilized in the intensive care unit without immediate complications. Short-term prognosis was cautiously optimistic, though long-term follow-up data were unavailable due to transfer to a tertiary care facility.

## Discussion

This case represents a rare instance of ANCA-associated vasculitis-related aortitis progressing to Stanford type A aortic dissection in GPA, notable for its rapid progression within 48 hours in a patient with newly diagnosed GPA, chronic low-dose prednisone use for Crohn’s disease, and well-controlled hypertension. Unlike prior reports often involving untreated inflammation or chronic disease, this case highlights aortitis progressing to dissection despite early steroid initiation and no prior vascular involvement, emphasizing the need for vigilant monitoring in atypical GPA presentations [7,9,10].

The mechanism involves PR3-ANCA-mediated neutrophil activation, leading to vascular injury, granuloma formation, and endothelial dysfunction [7,12]. In aortitis, PR3-ANCA triggers neutrophil degranulation, releasing cytokines (e.g., TNF-alpha and IL-1) and MMPs that degrade elastin and collagen in the aortic media, causing inflammatory weakening. Granulomatous inflammation, driven by T-cell recruitment, disrupts aortic wall integrity, while endothelial apoptosis promotes thrombosis and vessel fragility [8,12]. Severe hypertension (210/114 mmHg) acted as a mechanical trigger, causing a tear in the weakened aortic wall, as evidenced by initial CT aortic wall thickening progressing to CTA-confirmed dissection [7,8]. Clinical red flags, such as PR3-ANCA (4.3 AI, highly specific for GPA), c-ANCA (1:20, supportive), ESR (77 mm/hr), CRP (5.53 mg/dL), and intraoperative aortitis, reflect these mechanisms. The unusually low RDW (9.6%, normal: 11.5-14.5%) likely represents a lab artifact, as no clinical correlation (e.g., marrow suppression) exists, while elevated MPV (16.3 fL, normal: 7.5-11.5 fL) may indicate reactive thrombocytosis due to inflammation or vascular stress, though its prognostic significance in GPA is unclear [13]. Hydralazine, used for well-controlled hypertension, is associated with drug-induced lupus or vasculitis, but negative ANA and p-ANCA ruled out its relevance, with PR3-ANCA confirming GPA [14].

Distinguishing GPA-related aortitis from giant cell arteritis (GCA) and Takayasu arteritis (TAK) is critical, as it impacts treatment and prognosis. GCA, common in older adults, involves cranial artery inflammation and responds to high-dose corticosteroids and tocilizumab, while TAK, affecting younger patients, causes chronic stenosis and requires methotrexate or biologics [8,15]. GPA, driven by PR3-ANCA, follows a relapsing course, necessitating rituximab or cyclophosphamide, with aortic dissection requiring urgent surgical repair [2,7]. Histopathologically, GPA shows necrotizing granulomas and neutrophil infiltration, unlike GCA’s giant cells or TAK’s chronic fibrosis; PR3-ANCA positivity is a definitive marker for GPA, absent in GCA/TAK [8,12]. The absence of perivascular inflammation, vessel wall enhancement, or luminal narrowing on initial CT distinguished this case from classic large-vessel vasculitis, supporting GPA-related aortitis [8].

Diagnostic approach for suspected AAV-related aortitis

Serology

Order c-ANCA and PR3-ANCA to confirm GPA (PR3-ANCA, highly specific). Negative p-ANCA rules out MPA or drug-induced vasculitis [2].

Inflammatory Markers

Measure ESR and CRP to assess vasculitis activity. Elevated levels (e.g., ESR of 77 mm/hr and CRP at 5.53 mg/dL) support diagnosis [7].

Imaging

Use CTA for acute presentations to detect aortic wall thickening or dissection due to its high resolution and speed. MRI is preferred for chronic monitoring to assess vessel wall inflammation without radiation [11].

Biopsy

Consider aortic tissue biopsy during surgery to confirm necrotizing granulomas, as seen intraoperatively [8].

This approach prioritizes CTA in acute chest pain with suspected aortitis, followed by serologic confirmation, ensuring rapid diagnosis. The initial CT in this case showed aortic wall thickening without classic vasculitis signs, prompting a timely CTA on day two.

Treatment Considerations

For GPA with large-vessel involvement, rituximab is often preferred over cyclophosphamide due to its efficacy in inducing remission and better safety profile, particularly in non-renal disease [4,16]. Aortic dissection necessitates urgent surgical repair, as performed here (ascending aorta/hemiarch replacement), with immunosuppression (e.g., corticosteroids and rituximab) cautiously resumed post repair to manage GPA while minimizing infection risk [7]. Chronic low-dose prednisone (20 mg daily for Crohn’s disease) may have compounded vascular injury by inhibiting collagen synthesis, exacerbating aortic fragility, though high-dose methylprednisolone (1 g daily, initiated on day one) is unlikely to have caused dissection by day two due to the short timeframe and pre-existing aortitis [17]. Dissection alters the treatment plan by prioritizing surgical stabilization over immediate aggressive immunosuppression.

Prognosis

AAV-related aortic dissection carries a ~50% mortality rate without surgery, dropping to 20-30% with timely repair, though long-term complications include aortic insufficiency, aneurysm formation, and GPA relapse [7,9]. This case’s surgical success and postoperative stabilization suggest a favorable short-term outcome, though long-term follow-up is needed to monitor for relapse.

Case comparisons

Ayat et al. presented a 28-year-old with newly diagnosed GPA and Stanford type A dissection, driven by PR3-ANCA-mediated aortitis, which mirrors this case’s rapid progression but lacks chronic steroid exposure, highlighting prednisone’s unique role in our patient’s vessel fragility [10].

Niimi et al. presented a 38-year-old with GPA and aortic aneurysm rupture from untreated inflammation, which contrasts with this case’s dissection and chronic prednisone use, suggesting different inflammatory triggers [9].

Morshuis et al. presented a 53-year-old with MPA and fatal dissection due to misdiagnosis, as pneumonia emphasizes diagnostic challenges, unlike this case’s timely CTA and surgical success [7].

Approximately 16 cases of AAV-related aortic dissection have been reported, based on case reports rather than systematic reviews, with increasing recognition due to improved imaging (e.g., high-resolution CTA) [7,9,10]. This case underscores the importance of early detection and multidisciplinary management in mitigating the high morbidity and mortality of AAV-related aortic complications.

ANCA testing uses enzyme-linked immunosorbent assay (ELISA) and indirect immunofluorescence, with immunofluorescence offering greater sensitivity and ELISA providing higher specificity for specific antibodies. Imaging techniques for assessing large-vessel involvement include high-resolution contrast-enhanced MRI, CTA, Doppler sonography, PET with fluorodeoxyglucose (FDG), and digital subtraction angiography [11].

Treatment of AAV includes induction with corticosteroids or cyclophosphamide, after which maintenance is achieved with rituximab or azathioprine. Corticosteroids only reduce morbidity and mortality in large-vessel vasculitis, but they do not cure the disease nor prevent relapses [11]. Surgery is needed when a patient develops serious complications, such as an aortic aneurysm or dissection.

## Conclusions

This case highlights a rare instance of GPA-related aortitis progressing to Stanford type A aortic dissection within 48 hours, confirmed by PR3-ANCA (4.3 AI), c-ANCA (1:20), ESR (77 mm/hr), CRP (5.53 mg/dL), and CTA findings. The rapid progression underscores the need for early detection of large-vessel involvement in AAV using CTA and serologic testing. Misdiagnosis risks fatal outcomes, necessitating vigilant monitoring for atypical symptoms like sudden chest pain. Adaptable treatment strategies, including urgent surgical repair, rituximab-based immunosuppression, closer monitoring with serial imaging, and multidisciplinary care involving rheumatology, vascular surgery, and cardiology, are crucial for improving outcomes and reducing mortality in AAV-related aortic complications.
